# Radiotherapy Plus Chemotherapy Is Associated With Improved Survival Compared to Radiotherapy Alone in Patients With Primary Vaginal Carcinoma: A Retrospective SEER Study

**DOI:** 10.3389/fonc.2020.570933

**Published:** 2020-12-18

**Authors:** Wei-li Zhou, Yang-yang Yue

**Affiliations:** ^1^ Department of General Surgery, Shengjing Hospital of China Medical University, Shenyang, China; ^2^ Department of Health Management, Shengjing Hospital of China Medical University, Shenyang, China

**Keywords:** overall survival, squamous cell carcinoma, SEER, primary vaginal carcinoma, radiotherapy, chemotherapy, cancer-specific survival, adenocarcinoma

## Abstract

**Background:**

The efficacy of radiotherapy plus chemotherapy (RTCT) *versus* radiotherapy alone (RT) in the treatment of primary vaginal carcinoma has been controversial. We aimed to evaluate the up-to-date efficacy of RTCT on primary vaginal carcinoma in a real-world cohort.

**Methods:**

We performed a retrospective analysis in patients with primary vaginal carcinoma retrieved from the Surveillance, Epidemiology, and End Results Program database from 2004 to 2016. Kaplan–Meier survival curves were plotted and compared by the log-rank test. Inverse probability weighting (IPW)-adjusted multivariate Cox proportional hazards and Fine-Gray competing-risk model was applied.

**Results:**

Of the 1,813 qualified patients with primary vaginal carcinoma from 2004 to 2016, 1,137 underwent RTCT and 676 underwent RT. The median survival time was 34 months for the RT group and 63 months for the RTCT group. RTCT was significantly associated with improved overall survival (unadjusted HR = 0.71, 95% CI 0.62–0.82, p < 0.001; adjusted HR = 0.73, 95% CI 0.63–0.84, p < 0.001) and cancer-specific survival (unadjusted sHR = 0.81, 95% CI 0.69–0.95, p = 0.012; adjusted sHR = 0.81, 95% CI 0.69–0.96, p = 0.016). Age, histological type, tumor size, surgery, and FIGO stage were all independent prognostic factors for survival (p < 0.05 for all). Subgroup analysis demonstrated that RTCT was significantly associated with better survival in most subgroups, except for those with adenocarcinoma, tumor size <2 cm, or FIGO stage I. Moreover, sensitivity analysis did not alter the beneficial effects of RTCT.

**Conclusion:**

RTCT is significantly correlated with prolonged survival in patients with primary vaginal carcinoma. RTCT should be applied to most patients with primary vaginal carcinoma instead of RT alone, except for those with adenocarcinoma, tumor size <2 cm, or FIGO stage I.

## Introduction

Primary vaginal carcinoma is a rare malignancy that accounts for nearly 2% of all gynecologic cancer patients, with nearly 5,000 new patients diagnosed each year in the United States (1, 2). Vaginal carcinoma originates from the cells of the vagina and can be classified into the following histological types: 1) squamous cell carcinoma (SCC), accounting for 80–90% of cases; 2) adenocarcinoma (ADE), accounting for approximately 6% of cases; and 3) other histological types (Other, *e.g.*, melanoma and sarcoma). SCC and ADE have similar prognoses, but the prognoses differ from the prognosis of Other types (3, 4).

The most common treatment options for primary vaginal carcinoma are surgical resection, radiotherapy (including brachytherapy), and chemotherapy (5–11). However, the rarity of vaginal carcinoma makes it challenging to assess the efficacy of different treatment options applied to primary vaginal carcinoma patients. No randomized controlled trials comparing radiotherapy plus chemotherapy (RTCT) with radiotherapy alone (RT) in patients with primary vaginal carcinoma have been performed to date. Furthermore, there are currently no globally accepted guidelines regarding vaginal carcinoma treatment using RTCT (12).

Some studies maintain that chemotherapy should only be considered for patients with locally advanced vaginal cancer if patients are tolerant of chemotherapy. However, the use of chemotherapy in patients with early-stage vaginal carcinoma has been increasing since 1999 (13). The efficacy of RTCT has not been well defined in patients with primary vaginal carcinoma of differing biological characteristics, such as histological type, tumor size, and stage. Additionally, several published studies have reported conflicting conclusions. Thus, in this study, we sought to determine the efficacy of RTCT compared with RT in patients with biologically diverse primary vaginal cancer of diverse characteristics in a large real-world cohort.

## Patients and Methods

### Data Source and Study Population

The Surveillance, Epidemiology, and End Results (SEER) Program database of the National Cancer Institute was surveyed to obtain patients with primary vaginal carcinoma from 2004 to 2016. Patients with the 3rd Edition of the International Classification of Diseases for Oncology (ICD-O-3) site code of C52.9 were selected. Exclusion criteria were as follows: 1) patients with other tumors prior to primary vaginal carcinoma; 2) patients who were not treated with radiotherapy; 3) patients with survival time equal to 0 months; 4) patients with unknown surgical status.

The variables analyzed in this study included the year of diagnosis, age, race, marital status, histological type, pathological grade, tumor size, surgery, and International Federation of Gynecology and Obstetrics (FIGO) stage. Age was divided into two intervals separated by the median of age. The FIGO staging system is the most comprehensive and widely used staging system of gynecological cancers (14, 15). In this study, the FIGO stage was derived from the “CS extension codes” of the SEER database according to the FIGO staging system’s definition because these extension codes indicated the continued growth of a primary tumor to the adjacent tissue and organs (16). ICD-O-3 histology codes of 8,050–8,084 and 8,120–8,131 were considered as SCC, 8,140–8,389 as ADE, and all the remaining codes were considered to be Other (17).

The primary outcome of this study was overall survival (OS). The secondary outcome was cancer-specific survival (CSS), which was calculated on the basis of the number of vaginal cancer-related deaths. In contrast, death due to a reason other than vaginal cancer was considered as a competing risk during the Fine-Gray competing risk analysis.

An ethical review process was not needed because all the data of this study was obtained from the Surveillance, Epidemiology, and End Results (SEER) database, and we have signed the Data-use Agreement for the SEER 1975–2016 Research Data File. Patient information was retrieved from the SEER database, and the requirement for informed consent was waived.

### Statistical Analysis

The demographic and biological characteristics between patients treated with RTCT and RT were compared using the chi-square test. Inverse probability weighting (IPW) derived from a logit model was used to adjust for imbalances in variables between the two groups (18). Kaplan–Meier survival curves were plotted and compared using the log-rank test. We constructed a life table to estimate the 3-year, 5-year, and 10-year survival rate, with the 95% confidence interval (95% CI) calculated by Greenwood’s formula for the standard error (SE) of the estimates and quantile of the standard normal distribution. Multivariate Cox proportional hazard and Fine–Gray competing-risk models with and without IPW were applied to control confounding variables and to obtain the hazard ratio (HR) and the 95% CI of each variable. All variables were introduced into multivariate models without stepwise variable filtering through univariate models. Subgroup and sensitivity analyses were performed to comprehensively assess the efficacy of RTCT *vs*. RT comprehensively. A two-tailed *P*-value of less than 0.05 was considered to be statistically significant. All statistical analyses were performed in STATA 15.1 software (StataCorp, College Station, Texas).

## Results

### Demographic and Biological Characteristics

**Figure 1:**
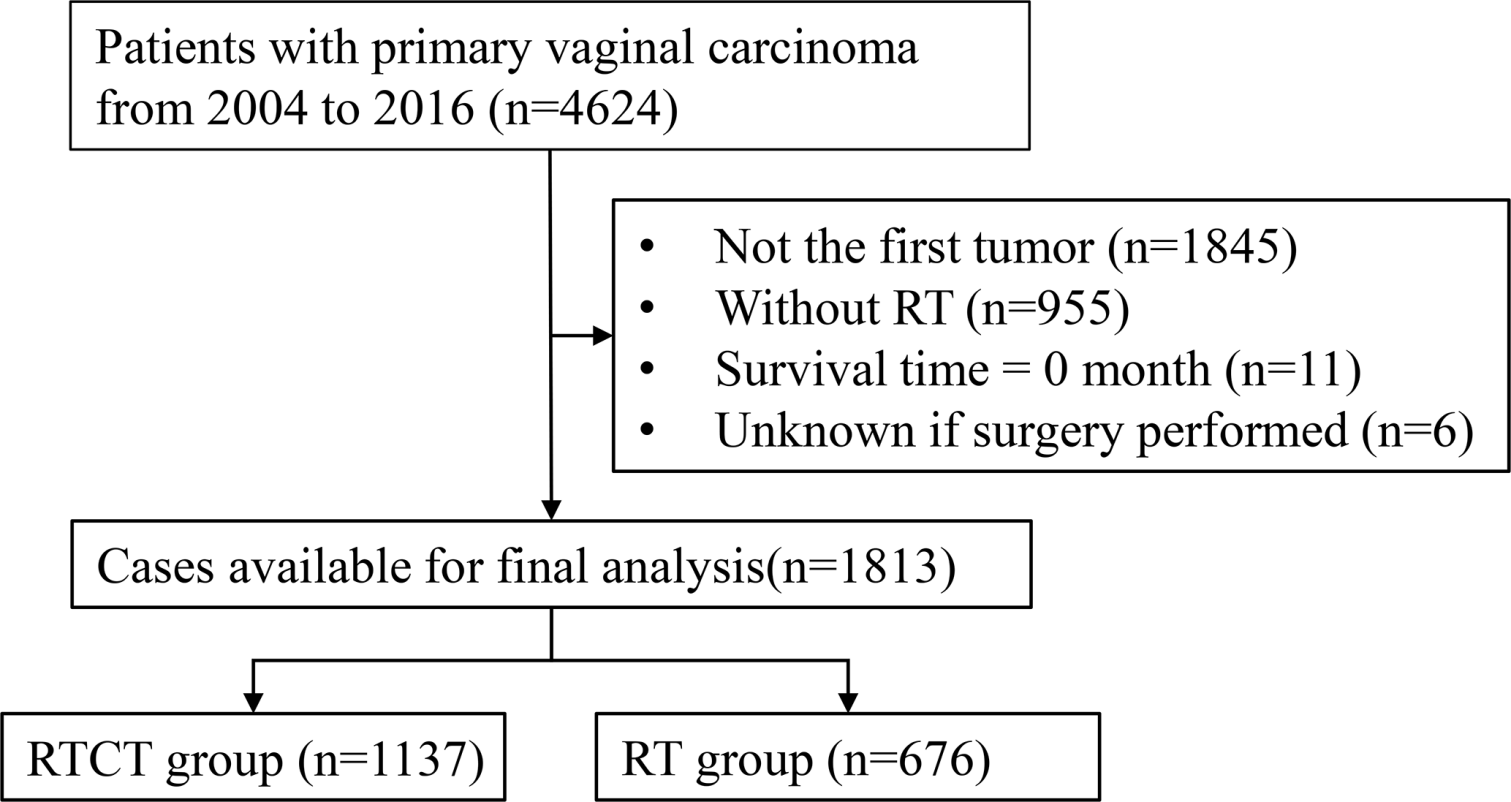
Flowchart of the patient selection procedure. RT, radiotherapy; RTCT, radiotherapy plus chemotherapy.

**Figure 2:**
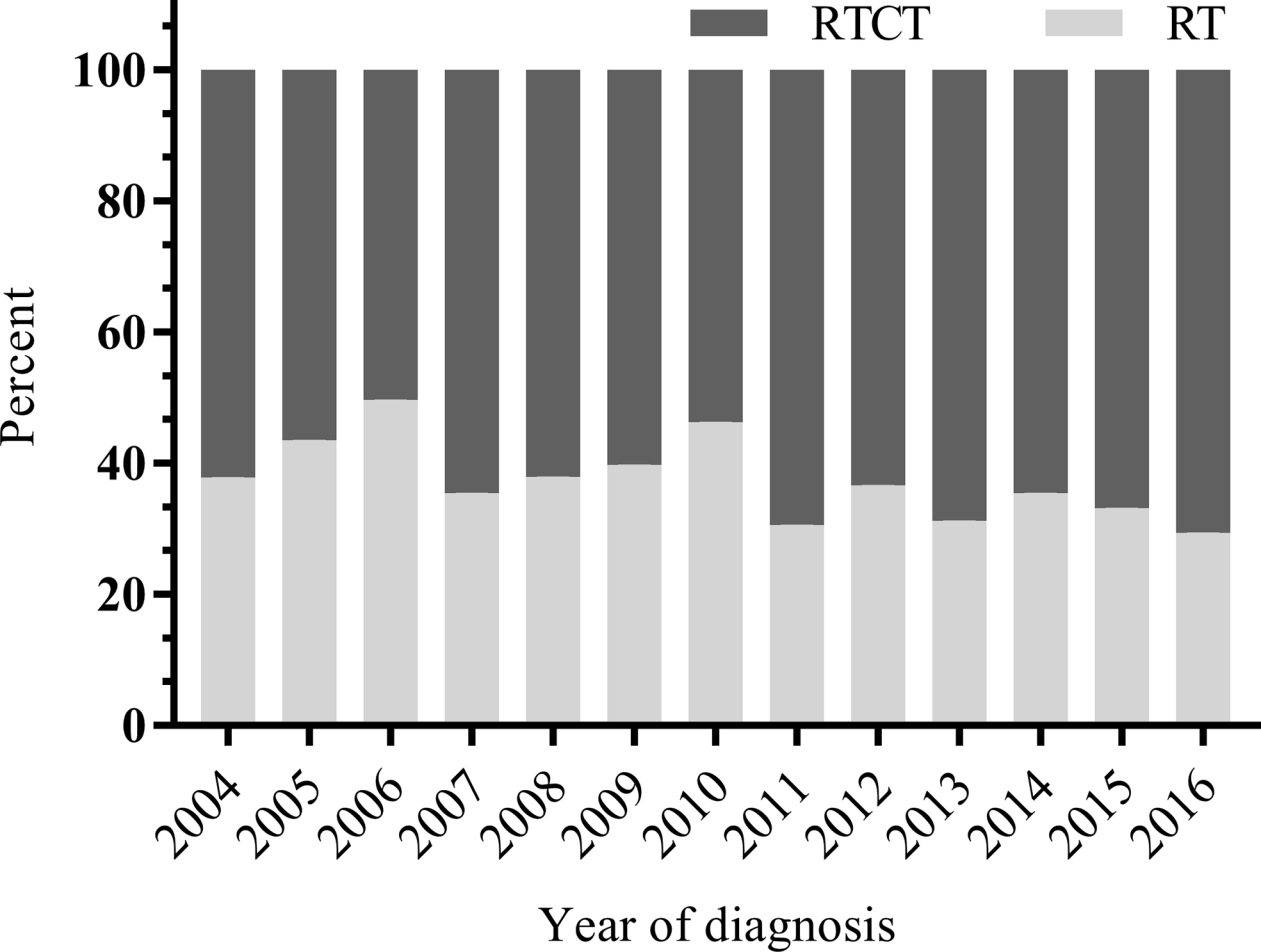
Distribution of RTCT *versus* RT by year from 2004 to 2016. On average, more than 60 percent of patients who underwent radiation received chemotherapy between 2004 and 2016, and the percentage increased slightly in recent years. RT, radiotherapy; RTCT, radiotherapy plus chemotherapy.

Of the 4,624 patients with primary vaginal cancer diagnosed between 2004 and 2016 identified from the SEER database, 1,813 (39.2%) were included in the final analysis. Of these 1,813 patients, 1,137 (62.7%) underwent RTCT, and 676 (37.3%) underwent RT alone. **Figure 1** displays the sample selection procedure. On average, more than 60 percent of patients who underwent radiation therapy from 2004 to 2016 also underwent chemotherapy, and the percentage increased slightly in recent years (**Figure 2**).

Patients treated with RTCT were more likely to be diagnosed between 2010 and 2016 (57.4% *vs.* 51.0%, p = 0.008). In the RTCT group, more patients were married or single status (44.1% *vs.* 35.2% and 18.2% *vs*. 13.3%, p < 0.001). More patients in the RTCT group were <65 years of age (56.9% *vs*. 35.2%, p < 0.001). Patients who underwent RTCT tended to have SCC (72.3% *vs.* 61.4%, p < 0.001), advanced pathological grade (grade II: 29.4% *vs*. 24.4%; grade III/IV: 36.1% *vs*. 33.1%, p < 0.001), larger tumor size (tumor size ≥4 cm: 40.8% *vs.* 30.5%, p < 0.001), and advanced FIGO stage (stage II: 37.9% *vs*. 29.1%; stage III/IV: 25.5% *vs*.19.1%, p < 0.001). Patients treated with RTCT also received surgery less often (23.9% *vs*. 36.2%, p < 0.001) (**Table 1**).

**Table 1:** Demographic and biological characteristics of patients with primary vaginal carcinoma from 2004 to 2016.

Characteristics	RT (%)	RTCT (%)	*P*-value
**Year of diagnosis**			0.008
2004–2009	331(49.0)	484(42.6)	
2010–2016	345(51.0)	653(57.4)	
**Age, years**			<0.001
<65	238(35.2)	647(56.9)	
≥65	438(64.8)	490(43.1)	
**Race**			0.085
Black	92(13.6)	173(15.2)	
Other	58(8.6)	68(6.0)	
White	526(77.8)	896(78.8)	
**Marital status**			<0.001
Married	238(35.2)	501(44.1)	
Single	90(13.3)	207(18.2)	
Divorced, separated, widowed	305(45.1)	385(33.9)	
Unknown	43(6.4)	44(3.9)	
**Histological type**			<0.001
SCC	415(61.4)	822(72.3)	
ADE	98(14.5)	168(14.8)	
Other	163(24.1)	147(12.9)	
**Pathological grade**			<0.001
I	62(9.2)	72(6.3)	
II	158(23.4)	334(29.4)	
III/IV	224(33.1)	410(36.1)	
Unknown	232(34.3)	321(28.2)	
**Tumor size, cm**			<0.001
<2	69(10.2)	54(4.7)	
2–4	144(21.3)	233(20.5)	
≥4	213(31.5)	464(40.8)	
Unknown	250(37.0)	386(33.9)	
**Surgery**			<0.001
No	431(63.8)	865(76.1)	
Yes	245(36.2)	272(23.9)	
**FIGO stage**			<0.001
I	274(40.5)	282(24.8)	
II	197(29.1)	431(37.9)	
III/IV	129(19.1)	290(25.5)	
Unknown	76(11.2)	134(11.8)	

RTCT, radiotherapy plus chemotherapy; RT, radiotherapy alone; SCC, squamous cell carcinoma; ADE, adenocarcinoma. FIGO, International Federation of Gynecology and Obstetrics.

### Prognostic Factors for Overall Survival and Cancer-Specific Survival

Multivariate Cox and Fine-Gray models with and without IPW demonstrated that patients treated with RTCT had significantly improved OS (unadjusted HR = 0.71, 95% CI: 0.62–0.82, p < 0.001; adjusted HR = 0.73, 95% CI: 0.63–0.84, p < 0.001) and CSS (unadjusted sHR = 0.81, 95% CI: 0.69–0.95, p = 0.012; adjusted sHR = 0.81, 95% CI: 0.69–0.96, p = 0.016) compared to that of patients treated with RT alone.

Age ≥65 years old, other histological types, tumor size ≥4 cm, and advanced FIGO stage were all independent prognostic factors for worse OS and CSS (p all <0.05). Moreover, patients who underwent surgery had prolonged OS and CSS (p all <0.05) compared with those who did not. Furthermore, divorced, separated, or widowed marital status was an adverse prognostic factor for OS (p < 0.01). Patients diagnosed between 2010 and 2016 had improved CSS (p < 0.05). However, we failed to identify a significant association between race or pathological grade with either OS or CSS (p all > 0.05) (**Tables 2** and **3**).

**Table 2:** Multivariate Cox proportional hazard models for overall survival with and without inverse probability weighting in patients with primary vaginal carcinoma.

Characteristics	Origin unweighted cohort		Inverse probability weighted cohort	
	HR (95% CI)	*P-*value	sHR (95% CI)	*P*-value
**Treatment**				
RT	Reference		Reference	
RTCT	0.71 (0.62–0.82)	<0.001	0.73 (0.63–0.84)	<0.001
**Year of diagnosis**				
2004–2009	Reference		Reference	
2010–2016	0.91 (0.80–1.05)	0.212	0.92 (0.79–1.07)	0.289
**Age, years**				
<65	Reference		Reference	
≥65	1.70 (1.47–1.97)	<0.001	1.93 (1.63–2.29)	<0.001
**Race**				
Black	Reference		Reference	
Other	0.77 (0.56–1.07)	0.121	0.74 (0.52–1.06)	0.102
White	0.97 (0.81–1.16)	0.724	0.96 (0.78–1.20)	0.744
**Marital status**				
Married	Reference		Reference	
Single	1.13 (0.92–1.38)	0.259	1.10 (0.86–1.40)	0.447
Divorced, separated or widowed	1.39 (1.19–1.63)	<0.001	1.37 (1.14–1.63)	0.001
Unknown	1.00 (0.72–1.39)	0.996	1.05 (0.75–1.49)	0.770
**Histological type**				
SCC	Reference		Reference	
ADE	0.90 (0.73–1.10)	0.299	0.82 (0.65–1.03)	0.086
Other	1.64 (1.36–1.98)	<0.001	1.62 (1.33–1.96)	<0.001
**Pathological grade**				
I	Reference		Reference	
II	0.99 (0.73–1.35)	0.954	0.91 (0.66–1.25)	0.543
III/IV	1.12 (0.83–1.51)	0.451	1.10 (0.81–1.49)	0.554
Unknown	0.95 (0.70–1.29)	0.747	0.97 (0.71–1.33)	0.845
**Tumor size, cm**				
<2	Reference		Reference	
2-4	1.01 (0.74–1.38)	0.961	1.02 (0.73–1.44)	0.889
≥4	1.41 (1.04–1.90)	0.026	1.39 (1.01–1.93)	0.045
Unknown	1.51 (1.11–2.04)	0.008	1.47 (1.06–2.04)	0.021
**Surgery**				
No	Reference		Reference	
Yes	0.65 (0.55–0.76)	<0.001	0.66 (0.55–0.79)	<0.001
**FIGO stage**				
I	Reference		Reference	
II	1.31 (1.10–1.55)	0.002	1.32 (1.09–1.58)	0.004
III/IV	2.05 (1.69–2.50)	<0.001	2.16 (1.76–2.65)	<0.001
Unknown	1.64 (1.23–2.18)	0.001	1.63 (1.19–2.23)	0.002

HR, hazard ratio; RTCT, radiotherapy plus chemotherapy; RT, radiotherapy alone; SCC, squamous cell carcinoma; ADE, adenocarcinoma. FIGO, International Federation of Gynecology and Obstetrics.

**Table 3:** Multivariate Fine-Gray competing-risks models for cancer-specific survival with and without inverse probability weighting in patients with primary vaginal carcinoma.

Characteristics	Origin unweighted cohort		Inverse probability weighted cohort	
	sHR (95% CI)	*P*-value	sHR (95% CI)	*P*-value
**Treatment**				
RT	Reference		Reference	
RTCT	0.81 (0.69–0.95)	0.012	0.81 (0.69–0.96)	0.016
**Year of diagnosis**				
2004–2009	Reference		Reference	
2010–2016	0.82 (0.70–0.96)	0.013	0.82 (0.69–0.98)	0.030
**Age, years**				
<65	Reference		Reference	
≥65	1.34 (1.14–1.58)	<0.001	1.53 (1.27–1.84)	<0.001
**Race**				
Black	Reference		Reference	
Other	0.99 (0.69–1.43)	0.976	0.90 (0.60–1.34)	0.598
White	1.10 (0.89–1.36)	0.372	1.07 (0.84–1.37)	0.590
**Marital status**				
Married	Reference		Reference	
Single	1.00 (0.79–1.25)	0.966	0.99 (0.76–1.30)	0.945
Divorced, separated or widowed	1.19 (1.00–1.42)	0.055	1.10 (0.90–1.34)	0.355
Unknown	0.77 (0.50–1.20)	0.249	0.75 (0.47–1.19)	0.216
**Histological type**				
SCC	Reference		Reference	
ADE	0.88 (0.70–1.10)	0.248	0.79 (0.61–1.02)	0.074
Other	1.72 (1.41–2.11)	<0.001	1.67 (1.35–2.07)	<0.001
**Pathological grade**				
I	Reference		Reference	
II	0.83 (0.60–1.13)	0.237	0.73 (0.52–1.03)	0.070
III/IV	0.94 (0.69–1.28)	0.704	0.91 (0.66–1.25)	0.544
Unknown	0.81 (0.59–1.11)	0.195	0.82 (0.59–1.13)	0.220
**Tumor size, cm**				
<2	Reference		Reference	
2–4	0.92 (0.63–1.33)	0.658	0.95 (0.63–1.43)	0.812
≥4	1.52 (1.06–2.17)	0.021	1.53 (1.05–2.24)	0.027
Unknown	1.39 (0.97–2.00)	0.072	1.34 (0.91–1.98)	0.136
**Surgery**				
No	Reference		Reference	
Yes	0.67 (0.56–0.81)	<0.001	0.69 (0.56–0.84)	<0.001
**FIGO stage**				
I	Reference		Reference	
II	1.42 (1.16–1.74)	0.001	1.44 (1.16–1.78)	0.001
III/IV	2.31 (1.85–2.88)	<0.001	2.42 (1.91–3.06)	<0.001
Unknown	1.70 (1.21–2.38)	0.002	1.73 (1.20–2.51)	0.004

sHR, subdistribution hazard ratio; RTCT, radiotherapy plus chemotherapy; RT, radiotherapy alone; SCC, squamous cell carcinoma; ADE, adenocarcinoma. FIGO, International Federation of Gynecology and Obstetrics.

### Survival Curve Analysis

**Figure 3:**
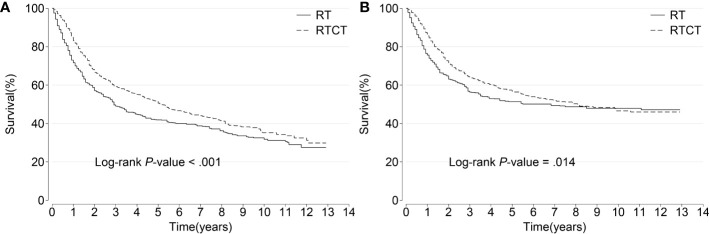
Kaplan–Meier curves of **(A)** overall and **(B)** cancer-specific survival among all patients. Patients who underwent RTCT had significantly prolonged overall and cancer-specific survival. RT, radiotherapy; RTCT, radiotherapy plus chemotherapy.

The median survival time was 34 months for the RT group and 63 months for the RTCT group. Overall, patients who received RTCT had prolonged OS (p < 0.001, log-rank test) and CSS (p = 0.014) (**Figure 3**).

**Table 4** shows the 3-year, 5-year, and 10-year OS and CSS rates within each subgroup stratified by histological type and FIGO stage. They demonstrate that patients treated with RTCT survived longer than those treated with RT alone in most of the subgroups.

**Table 4:** The 3-year, 5-year, and 10-year OS and CSS rates in subgroups stratified by histological type and FIGO stage.

Subgroup	Interval	RT		RTCT		*P*-value
		Survival % (95% CI)	Deaths/Total	Survival % (95% CI)	Deaths/Total	
**OS**						
**Histological type**						
SCC						<0.001
	3-years	50.4(45.3–55.3)	192/415	60.4(56.7–63.8)	289/822	
	5-years	40.8(35.7–45.8)	30/168	52.9(49.0–56.7)	38/349	
	10-years	31.7(26.4–37.1)	19/116	38.0(33.4–42.5)	47/231	
ADE						0.222
	3-years	66.6(55.9–75.3)	30/98	65.5(57.5–72.5)	53/168	
	5-years	60.9(49.7–70.4)	4/52	56.7(48.0–64.4)	10/87	
	10-years	52.7(40.4–63.6)	4/38	39.2(29.2–49.0)	12/51	
Other						0.001
	3-years	34.2(26.7–41.7)	100/163	52.9(44.2–61.0)	63/147	
	5-years	29.3(22.0–37.0)	5/41	43.3(34.4–51.8)	10/58	
	10-years	18.4(11.5–26.6)	8/25	30.7(21.4–40.4)	8/42	
**FIGO stage**						
I						0.614
	3-years	65.3(59.1–70.7)	89/274	68.2(62.2–73.5)	82/282	
	5-years	57.2(50.8–63.2)	17/150	61.3(54.8–67.1)	14/153	
	10-years	45.5(38.4–52.4)	17/110	44.3(36.6–51.8)	21/108	
II						<0.001
	3-years	45.8(38.6–52.8)	102/197	61.2(56.2–65.8)	154/431	
	5-years	34.8(27.8–41.9)	17/78	53.0(47.8–58.0)	25/210	
	10-years	26.1(19.1–33.5)	9/46	39.9(34.0–45.8)	25/138	
III/IV						<0.001
	3-years	21.9(15.1–29.4)	98/129	49.3(43.2–55.1)	138/290	
	5-years	17.9(11.6–25.3)	4/24	41.1(35.0–47.1)	17/117	
	10-years	13.7(7.95–21.2)	3/16	24.2(17.9–31.0)	21/71	
Unknown						0.006
	3-years	44.0(31.2–56.1)	33/76	65.3(54.5–74.2)	31/134	
	5-years	38.8(24.4–53.1)	1/9	54.0(36.3–68.6)	2/14	
	10-years	21.6(6.25–42.8)	2/7	54.0(36.3–68.6)	0/7	
**CSS**						
**Histological type**						
SCC						0.119
	3-years	58.8(53.6–63.6)	151/415	64.9(61.3–68.3)	249/822	
	5-years	53.3(47.8–58.4)	14/168	58.9(55.0–62.6)	28/349	
	10-years	51.2(45.5–56.7)	3/116	49.8(45.0–54.3)	24/231	
ADE						0.054
	3-years	76.4(65.9–84.1)	20/98	71.1(63.1–77.7)	43/168	
	5-years	71.5(60.1–80.2)	3/52	62.4(53.5–70.1)	9/87	
	10-years	66.5(53.6–76.5)	2/38	44.5(33.7–54.8)	11/51	
Other						0.003
	3-years	39.2(31.3–47.0)	89/163	57.6(48.7–65.6)	55/147	
	5-years	34.7(26.7–42.8)	4/41	47.2(37.8–55.9)	10/58	
	10-years	31.0(22.5–39.8)	2/25	38.1(27.9–48.2)	5/42	
**FIGO stage**						
I						0.744
	3-years	72.3(66.3–77.5)	68/274	74.0(68.0–78.9)	65/282	
	5-years	67.5(61.0–73.1)	9/150	68.0(61.5–73.6)	11/153	
	10-years	64.8(57.9–70.9)	3/110	57.4(49.4–64.7)	11/108	
II						0.019
	3-years	54.9(47.3–61.9)	80/197	65.6(60.6–70.1)	133/431	
	5-years	48.2(40.3–55.7)	8/78	59.1(53.8–64.0)	18/210	
	10-years	45.3(36.8–53.3)	2/46	48.8(42.5–54.8)	17/138	
III/IV						<0.001
	3-years	28.0(20.3–36.2)	86/129	53.3(47.1–59.0)	124/290	
	5-years	24.1(16.5–32.4)	3/24	44.9(38.5–51.1)	16/117	
	10-years	22.1(14.4–30.8)	1/16	33.3(26.1–40.7)	12/71	
Unknown						0.024
	3-years	53.1(39.2–65.2)	26/76	71.1(60.3–79.4)	25/134	
	5-years	46.9(30.1–62.0)	1/9	58.7(39.4–73.7)	2/14	
	10-years	35.1(13.9–57.5)	1/7	58.7(39.4–73.7)	0/7	

RTCT, radiotherapy plus chemotherapy; RT, radiotherapy alone; SCC, squamous cell carcinoma; ADE, adenocarcinoma. FIGO, International Federation of Gynecology and Obstetrics.

Kaplan-Meier plots demonstrate the beneficial effects of RTCT compared to those of RT alone in nearly all of the subgroups stratified by tumor size and FIGO stage, except for the tumor size <2 cm and FIGO stage I subgroups (**Supplementary Figures 1**–**4**).

### Subgroup and Sensitivity Analysis

To gain insight into the efficacy of RTCT within particular subgroups, we performed IPW-adjusted Cox proportional hazard models and Fine-Gray competing risk models within each subgroup stratified by histological type, tumor size, and FIGO stage. We found that the improved OS and CSS were attributable to RTCT in most subsets, except for a worse CSS related to RTCT in the tumor size <2 cm group (adjusted HR = 3.17, 95% CI: 1.15–8.77, p = 0.026) (**Table 5**).

**Table 5:** Hazard ratios of RTCT *versus* RT extracted from the inverse probability weighted multivariate Cox proportional hazard and Fine–Gray competing-risks models within each subgroup categorized by histological type, tumor size, and FIGO stage.

Characteristics	Overall survival		Cancer-specific survival	
	HR (95% CI)	*P*-value	sHR (95% CI)	*P*-value
**Histological type**				
SCC	0.72 (0.60–0.87)	0.001	0.81 (0.65–1.00)	0.050
ADE	0.87 (0.53–1.42)	0.574	1.02 (0.57–1.83)	0.939
Other	0.60 (0.43–0.83)	0.002	0.65 (0.46–0.92)	0.016
**Tumor size, cm**				
<2	1.60 (0.72–3.55)	0.246	3.17 (1.15–8.77)	0.026
2–4	0.70 (0.50–0.98)	0.040	0.85 (0.56–1.29)	0.441
≥4	0.68 (0.55–0.86)	0.001	0.75 (0.58–0.96)	0.021
Unknown	0.71 (0.57–0.90)	0.005	0.81 (0.61–1.07)	0.141
**FIGO stage**				
I	0.99 (0.75–1.30)	0.933	1.10 (0.78–1.53)	0.594
II	0.78 (0.61–0.98)	0.033	0.92 (0.70–1.22)	0.581
III/IV	0.50 (0.39–0.64)	<0.001	0.54 (0.40–0.73)	<0.001
Unknown	0.48 (0.26–0.89)	0.019	0.55 (0.26–1.16)	0.117

HR, hazard ratio; sHR, subdistribution hazard ratio; RTCT, radiotherapy plus chemotherapy; RT, radiotherapy alone; SCC, squamous cell carcinoma; ADE, adenocarcinoma. FIGO, International Federation of Gynecology and Obstetrics.

Considering that some unbalanced factors might confound results, we calculated new IPW among patients with tumor size <2 cm based on year of diagnosis, age, race, marital status, histological type, pathological grade, surgery, and FIGO stage, and carried out IPW-adjusted multivariate Cox hazard and Fine–Gray compete-risk regression analyses. The negative association between RTCT and survival remained in the tumor size <2 cm group.

Considering that tumor size and FIGO stage were high-risk factors and had a high proportion of unknown values, we carried out a sensitivity analysis. We considered three extreme scenarios, in which tumors with unknown size were all classified into <2 cm, 2–4 cm, or ≥4 cm subgroups. In those three scenarios, multivariate Cox proportional hazard and Fine–Gray competing risk models were both carried out and revealed that the beneficial effect of RTCT compared to that of RT alone had not changed. Moreover, a similar sensitivity analysis performed on the FIGO stage did not alter the superiority of RTCT either.

## Discussion

In this real-world cohort study, our critical finding was that patients with primary vaginal carcinoma could benefit more from RTCT than from RT alone, both overall and within particular subsets, except for those with adenocarcinoma, tumor size <2 cm, and FIGO stage I. Based on a large real-world SEER cohort from the United States and adjusting for a series of confounding factors, this study added supporting evidence for the superior efficacy of RTCT in the treatment of primary vaginal carcinoma.

The idea of applying RTCT to the management of vaginal carcinoma was thought to be inspired by and extrapolated from the impressive outcome achieved by RTCT used in the treatment of cervical and vulvar cancer, due to the similar etiology, histological type, and patterns of disease progression of these three cancer types. However, vaginal carcinoma has higher mortality than that of either cervical cancer or vulvar cancer (19). Given the rarity of vaginal carcinoma, randomized controlled trials evaluating the efficacy of RTCT *versus* RT are lacking and, to some extent, are not even feasible. Instead, clinicians have relied upon the results of retrospective observational studies to obtain evidence about the application of RTCT to the management of vaginal carcinoma.

Chemotherapy has increasingly been suggested in addition to RT in patients with vaginal carcinoma. Moreover, RTCT was shown to be well-tolerated and had limited toxicity in a population of patients with cervical and endometrial cancer (20, 21). However, little is known about chemotherapy for primary vaginal cancer patients, especially within particular subsets. After IPW adjustment, we found that RTCT led to significantly prolonged survival in patients with primary vaginal cancer compared with RT alone; these findings were consistent with two published studies with small sample sizes (8, 22) as well as a large retrospective study using the National Cancer Database (NCDB) (23). The NCDB study described some crucial variables, such as the year of diagnosis, tumor size, and surgery; however, those factors were not introduced into the survival analysis, so the impact of those confounding factors was not controlled for.

In contrast to our findings, some previously published studies have argued that concurrent chemoradiotherapy only facilitated improvement in local control improvement and did not prolong survival time (7, 24–27). Those studies were limited by small sample sizes of fewer than 80 patients and were therefore vulnerable to selection bias. Additionally, a study evaluating the efficacy of RTCT on vaginal cancer that had a sample size of 326 patients from SEER-Medicare failed to identify a survival benefit for RTCT (13). However, only 27 (7.5%) patients were treated with RTCT, which might not have been enough to show statistical significance. Our study, with a large sample size of 1,813 patients and a more well-chosen representative cohort, provided the power to detect the survival benefit afforded by RTCT using the SEER database. Notably, this study controlled for several factors, including the year of diagnosis, histological type, tumor size, surgery, and FIGO stage, when investigating the impact of RTCT on OS and CSS. Our findings extended those of the NCDB study and characterized the efficacy of RTCT in particular subsets (23).

In the subgroup analysis, treatment with RTCT had superior survival compared to treatment with RT alone for patients with SCC and other histological types. However, for those with ADE, the survival difference between the two groups did not reach statistical significance, likely due to the small sample size, although vaginal ADE has been reported to have a higher incidence of local recurrence and frequently metastasizes to the lung and supraclavicular and pelvic nodes (14, 16, 28, 29). Studies with larger size are warranted to address this. Large tumor size was an independent inferior prognostic factor for survival, consistent with the findings of other studies (11, 22, 23, 26, 27, 30). We identified a significantly improved survival outcome attributable to RTCT among patients with tumors sized 2–4 and ≥4 cm. That may be because large tumors are more likely to intrude into the surrounding muscles, lymph nodes, connective tissues, and distant sites, and therefore, RTCT demonstrated better control over cancer progression and led to more significant benefits (27, 31).

Notably, our study found that among patients with tumor size <2 cm, RTCT was associated with inferior OS and DSS compared to RT alone, even in the sensitivity analysis with all other factors controlled for. This may be because RTCT provides more drawbacks than benefits for patients with tumors measuring <2 cm, although the possibility that the small sample size led to the insignificance efficacy of RTCT cannot be ruled out. Further studies with larger sample sizes are needed to confirm these findings and address the reasons. For clinicians, more care should be taken when applying RTCT in tumors sized <2 cm. Additionally, after adjusting for several factors, we found that divorced, separated, or widowed women had worse OS than married women, which confirms the benefits of marriage on survival found by previous studies regarding vulvar, ovarian, endometrial, and breast cancers (32–35). The most likely reason for the survival benefit from marriage is that divorced, separated, or widowed patients have worse adherence to prescribed treatments than married patients. Due to lacking a partner to share the emotional burden and to get appropriate social support, divorced, separated, or widowed patients commonly experience more distress, depression, and anxiety than married counterparts, which probably leads to their poor adherence to treatment (36). Therefore, physicians should consider closer observation in divorced, separated, or widowed patients to screen for their negative emotions, and, if the symptoms are identified, refer them to mental health specialists to promote and maximize their adherence to treatment. Our study indicates the importance of psychosocial support-based intervention on the survival of vaginal cancer patients.

To our knowledge, this is the second-largest study conducted to date, consisting of 1,813 patients with primary vaginal carcinoma. Further, this study controlled for a diverse array of factors, including the year of diagnosis, age, race, marital status, histological type, pathological grade, tumor size, surgery, and FIGO stage, further strengthening our findings and broadening their generalizability. Since it can be argued that the receipt of RTCT was affected by some factors, such as the year of diagnosis, tumor size, surgery, and FIGO stage, we additionally performed IPW-adjusted multivariate Cox proportional hazard models and Fine-Gray competing-risk models to address the imbalances in variables between RTCT and RT groups, which further enhances the robustness of our conclusions. The positive association between RTCT and survival remained consistent both with and without IPW adjustment. This study is one of the few studies to introduce IPW into the survival factor identification procedure as well as Kaplan–Meier survival curve analysis, and simultaneously take both OS and CSS into account in evaluating the efficacy of RTCT *versus* RT for vaginal cancer, and the findings persuasively support our conclusions.

Our study has the following limitations. 1) the SEER database does not contain granular details such as the initiation and termination of radiotherapy and chemotherapy, so we could not identify the sequence in which radiation and chemotherapy were applied. Thus, we could not determine whether chemotherapy was concurrent or adjuvant; 2) this is a retrospective study of patients treated over a long period of time. Therefore, there may be missing confounders, which could introduce bias into our findings; 3) advances in imaging, diagnostic, and treatment techniques might also lead to bias. Although we introduced the year of diagnosis in the multivariate analysis, this may not have been adequate to completely control for their effect; 4) although tumor location is an important prognostic factor, this information is missing in the SEER database; therefore, similar to the NCDB study, the detailed location of the tumor within the vagina was not adjusted for.

Despite these limitations, this is a real-world cohort study with a large sample size that elucidated the efficacy of RTCT in patients with primary vaginal carcinoma and characterized the efficacy within particular subsets. Our current study contributes to understanding the application of CTRT on this rare cancer and adds to the sparse literature on primary vaginal carcinoma. Our findings will make oncologists consider the efficacy of RTCT in patients with primary vaginal carcinoma subclassified by histological type, tumor size, surgery, and FIGO stage.

## Conclusion

RTCT resulted in significantly prolonged survival in patients with primary vaginal carcinoma, except for those with adenocarcinoma, tumor size <2 cm, and FIGO stage I. The application of RTCT should be preferentially considered by clinicians for the majority of patients with primary vaginal carcinoma.

## Data Availability Statement

Publicly available datasets were analyzed in this study. This data can be found here: https://seer.cancer.gov/.

## Ethics Statement

Ethical review and approval was not required for the study on human participants in accordance with the local legislation and institutional requirements. Written informed consent for participation was not required for this study in accordance with the national legislation and the institutional requirements.

## Author Contributions

W-LZ: conceptualization, data curation, formal analyses, supervision, writing-original draft preparation. Y-YY: conceptualization, methodology, software, validation, visualization, supervision, writing-reviewing, and editing. All authors contributed to the article and approved the submitted version.

## Conflict of Interest

The authors declare that the research was conducted in the absence of any commercial or financial relationships that could be construed as a potential conflict of interest.

## Supplementary Material

Supplementary Figure 1 Kaplan–Meier curves of overall survival by tumor size, **(A)** <2 cm, **(B)** 2–4 cm, **(C)** ≥4 cm, **(D)** unknown. The overall survival was superior among patients who received RTCT within tumor size 2–4 cm, ≥4 cm, and unknown subgroups. However, non-significantly worse overall survival was observed with RTCT within the tumor size <2 cm subgroup. RT, radiotherapy; RTCT, radiotherapy plus chemotherapy.

Supplementary Figure 2 Kaplan–Meier curves of overall survival by FIGO stage, **(A)** stage I, **(B)** stage II, **(C)** stage III/IV, **(D)** stage unknown. Patients who underwent RTCT had prolonged overall survival in the FIGO stages II, III/IV, and unknown subgroups. Nevertheless, a non-significant reduction in overall survival was observed with RTCT in the FIGO stage I subgroup (p = 0.385 and p = 0.223, respectively). RT, radiotherapy; RTCT, radiotherapy plus chemotherapy; FIGO, International Federation of Gynecology and Obstetrics.

Supplementary Figure 3 Kaplan–Meier curves of cancer-specific survival by tumor size, **(A)** <2 cm, **(B)** 2–4 cm, **(C)** ≥4 cm, **(D)** unknown. The cancer-specific survival was superior among patients who received RTCT in the tumor size 2–4 cm, ≥4 cm, and unknown subgroups. However, non-significantly worse cancer-specific survival was observed with RTCT in tumor size <2 cm subgroup. RT, radiotherapy; RTCT, radiotherapy plus chemotherapy.

Supplementary Figure 4 Kaplan–Meier curves of cancer-specific survival by FIGO stage, **(A)** stage I, **(B)** stage II, **(C)** stage III/IV, **(D)** stage unknown. Patients who underwent RTCT had prolonged cancer-specific survival in FIGO stages II, III/IV, and Unknown subgroups. Nevertheless, a non-significant reduction in cancer-specific survival was observed with RTCT in the FIGO stage I subgroup. RT, radiotherapy; RTCT, radiotherapy plus chemotherapy; FIGO, International Federation of Gynecology and Obstetrics.
