# Supplementary figures and images for: Radiotherapy Plus Chemotherapy Is Associated With Improved Survival Compared to Radiotherapy Alone in Patients With Primary Vaginal Carcinoma: A Retrospective SEER Study
Source: Front Oncol. 2020 Dec 18;10:570933. doi: 10.3389/fonc.2020.570933 (PMC7775586; doi:10.3389/fonc.2020.570933)

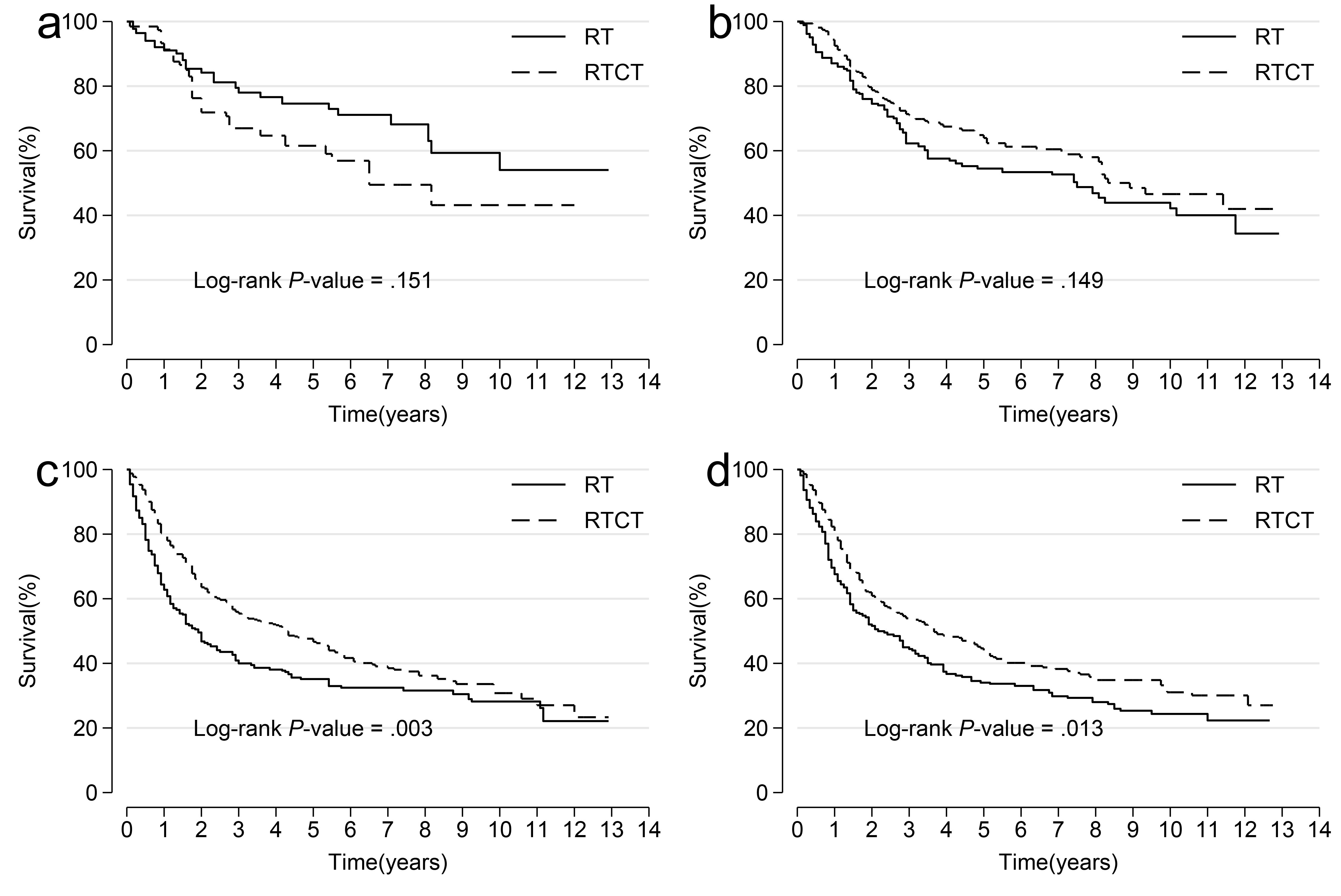

Supplement: Supplementary Figure 1 — Kaplan–Meier curves of overall survival by tumor size, (A) <2 cm, (B) 2–4 cm, (C) ≥4 cm, (D) unknown. The overall survival was superior among patients who received RTCT within tumor size 2–4 cm, ≥4 cm, and unknown subgroups. However, non-significantly worse overall survival was observed with RTCT within the tumor size <2 cm subgroup. RT, radiotherapy; RTCT, radiotherapy plus chemotherapy. [file Image_1.tif]

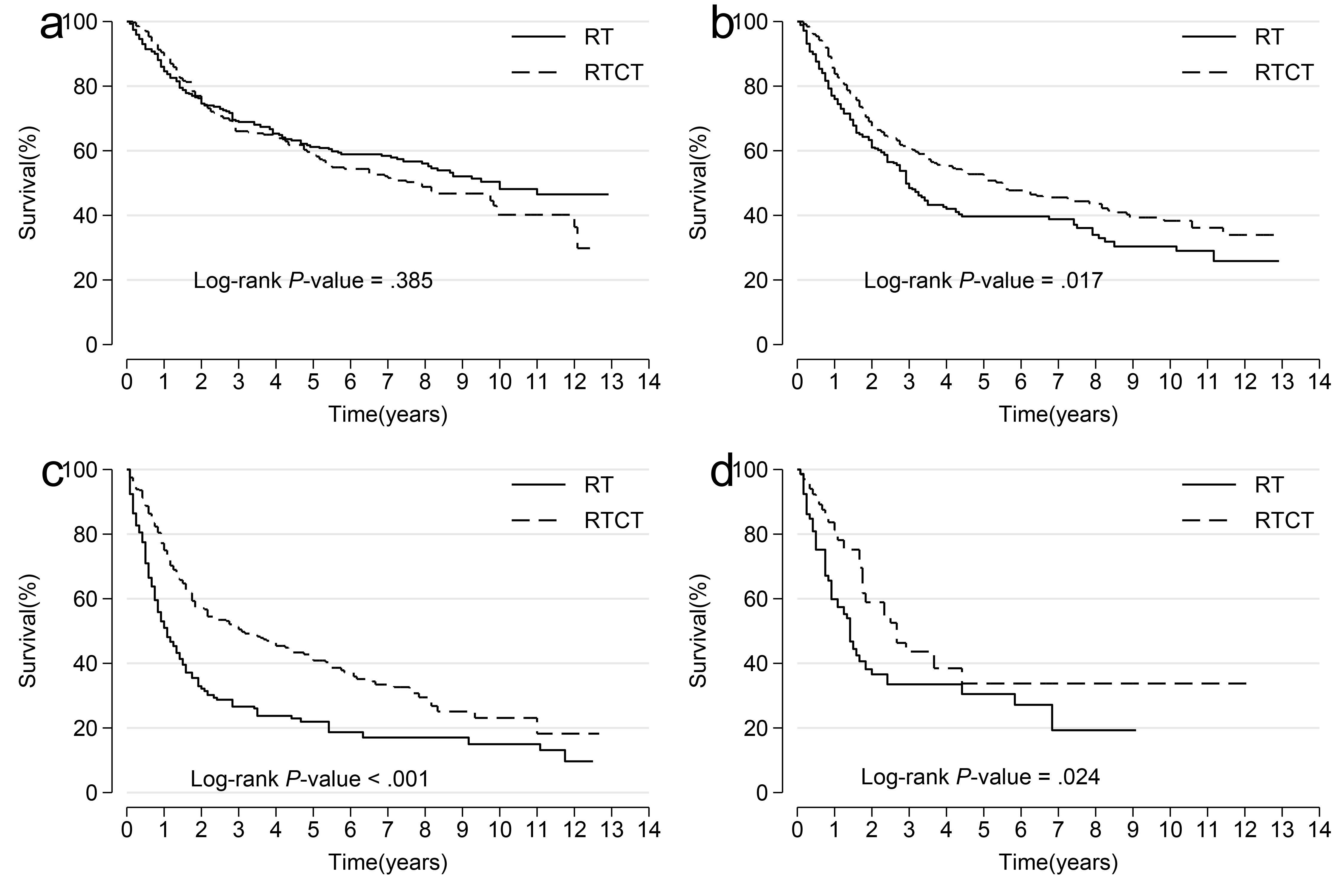

Supplement: Supplementary Figure 2 — Kaplan–Meier curves of overall survival by FIGO stage, (A) stage I, (B) stage II, (C) stage III/IV, (D) stage unknown. Patients who underwent RTCT had prolonged overall survival in the FIGO stages II, III/IV, and unknown subgroups. Nevertheless, a non-significant reduction in overall survival was observed with RTCT in the FIGO stage I subgroup (p = 0.385 and p = 0.223, respectively). RT, radiotherapy; RTCT, radiotherapy plus chemotherapy; FIGO, International Federation of Gynecology and Obstetrics. [file Image_2.tif]

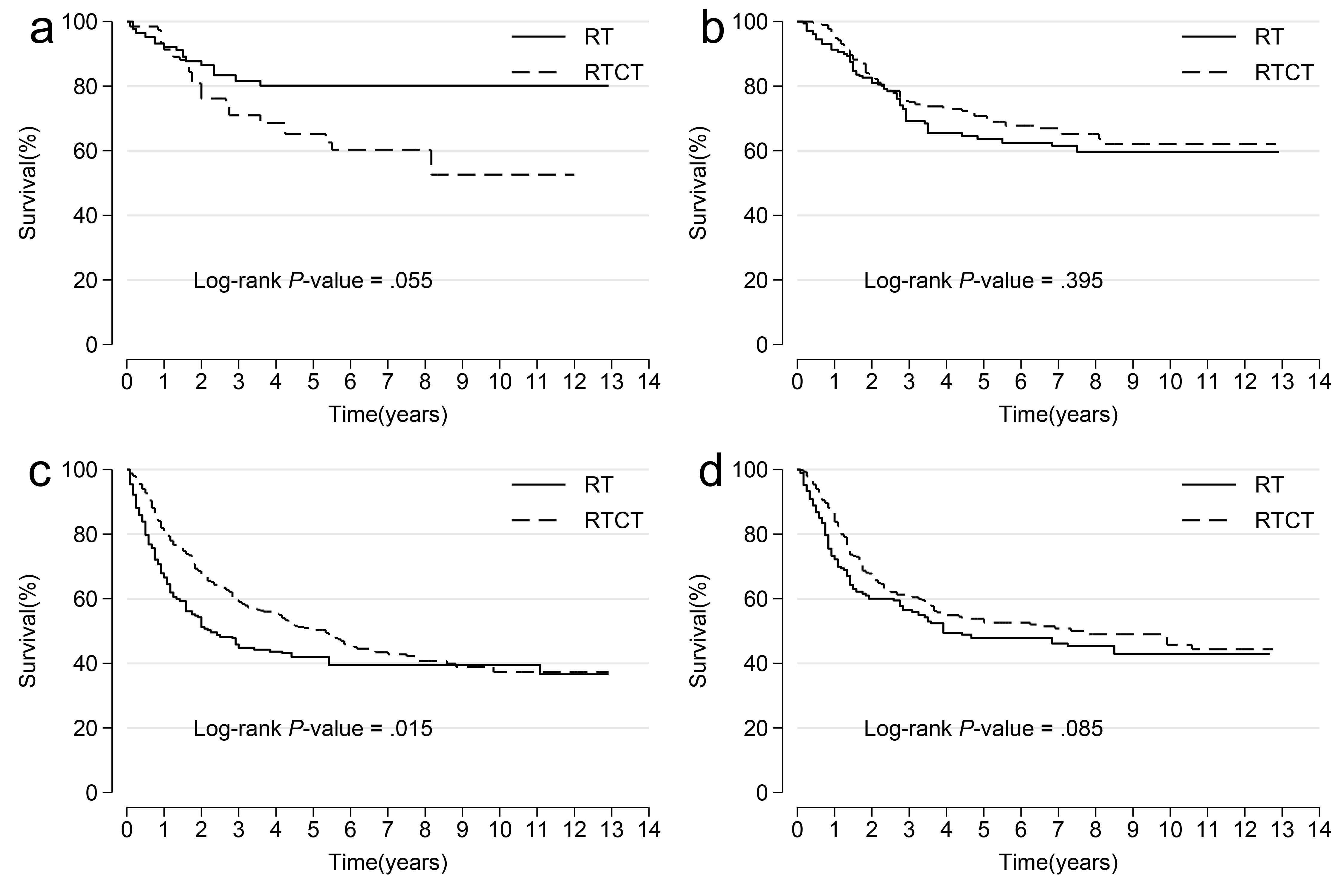

Supplement: Supplementary Figure 3 — Kaplan–Meier curves of cancer-specific survival by tumor size, (A) <2 cm, (B) 2–4 cm, (C) ≥4 cm, (D) unknown. The cancer-specific survival was superior among patients who received RTCT in the tumor size 2–4 cm, ≥4 cm, and unknown subgroups. However, non-significantly worse cancer-specific survival was observed with RTCT in tumor size <2 cm subgroup. RT, radiotherapy; RTCT, radiotherapy plus chemotherapy. [file Image_3.tif]

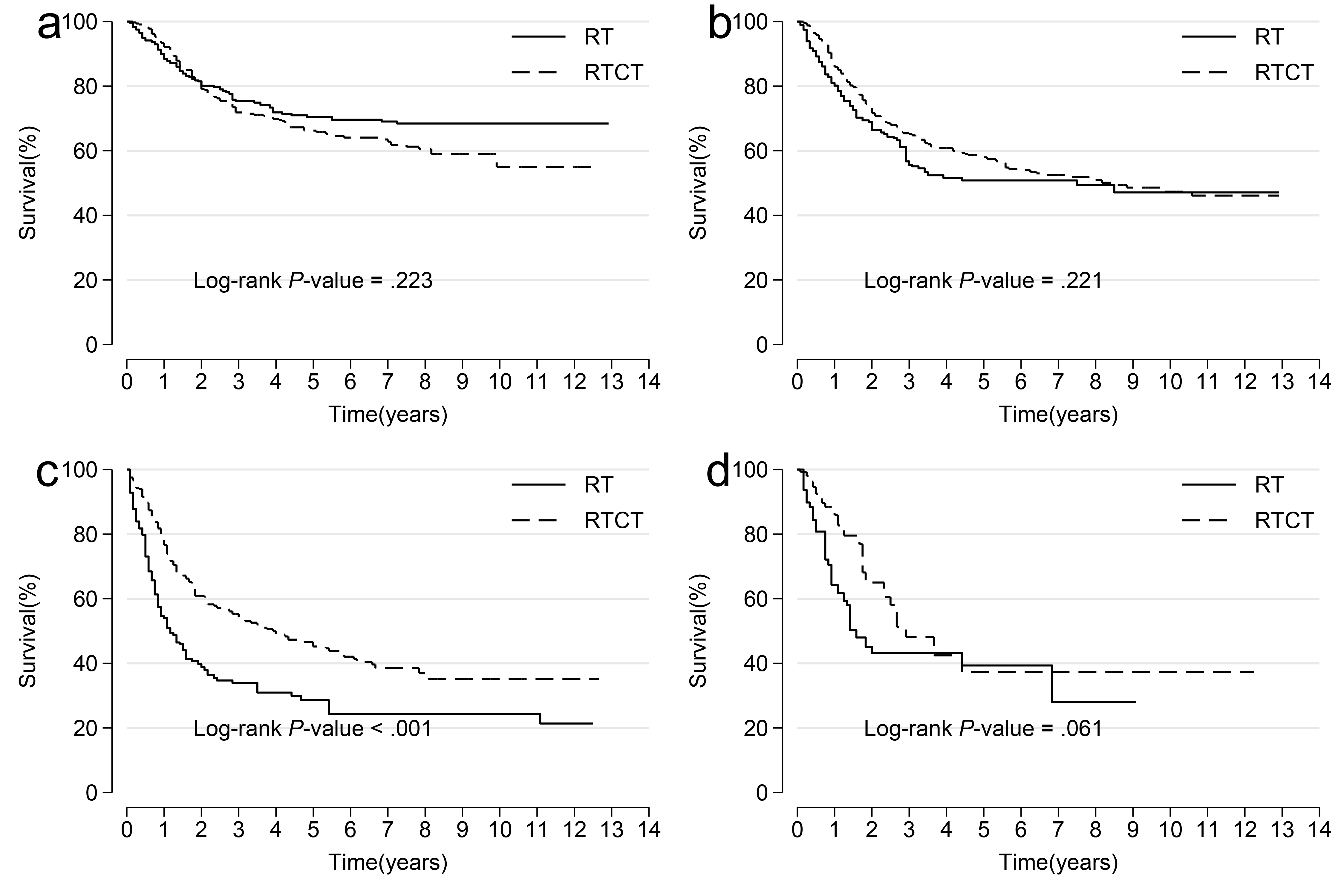

Supplement: Supplementary Figure 4 — Kaplan–Meier curves of cancer-specific survival by FIGO stage, (A) stage I, (B) stage II, (C) stage III/IV, (D) stage unknown. Patients who underwent RTCT had prolonged cancer-specific survival in FIGO stages II, III/IV, and Unknown subgroups. Nevertheless, a non-significant reduction in cancer-specific survival was observed with RTCT in the FIGO stage I subgroup. RT, radiotherapy; RTCT, radiotherapy plus chemotherapy; FIGO, International Federation of Gynecology and Obstetrics. [file Image_4.tif]
